# The Roles of the Amyloid Beta Monomers in Physiological and Pathological Conditions

**DOI:** 10.3390/biomedicines11051411

**Published:** 2023-05-10

**Authors:** Thomas Gabriel Schreiner, Oliver Daniel Schreiner, Maricel Adam, Bogdan Ovidiu Popescu

**Affiliations:** 1 Faculty of Medicine, University of Medicine and Pharmacy “Grigore T. Popa”, 700115 Iași, Romania; schreiner.oliver-daniel@email.umfiasi.ro; 2 Faculty of Electrical Engineering and Information Technology, Gheorghe Asachi Technical University of Iasi, 21–23 Professor Dimitrie Mangeron Blvd., 700050 Iasi, Romania; adamm@tuiasi.ro; 3 Faculty of Medicine, University of Medicine and Pharmacy “Carol Davila”, 020021 Bucharest, Romania; bogdan.popescu@umfcd.ro; 4 Medical Oncology Department, Regional Institute of Oncology, 700483 Iași, Romania; 5 Neurology Department, Colentina Clinical Hospital, 020125 Bucharest, Romania; 6 Laboratory of Cell Biology, Neurosciences and Experimental Myology, Victor Babes National Institute of Pathology, 050096 Bucharest, Romania

**Keywords:** Alzheimer’s disease, amyloid beta, monomer, amyloidogenic pathway, biomarker

## Abstract

Amyloid beta peptide is an important biomarker in Alzheimer’s disease, with the amyloidogenic hypothesis as one of the central hypotheses trying to explain this type of dementia. Despite numerous studies, the etiology of Alzheimer’s disease remains incompletely known, as the pathological accumulation of amyloid beta aggregates cannot fully explain the complex clinical picture of the disease. Or, for the development of effective therapies, it is mandatory to understand the roles of amyloid beta at the brain level, from its initial monomeric stage prior to aggregation in the form of senile plaques. In this sense, this review aims to bring new, clinically relevant data on a subject intensely debated in the literature in the last years. In the first part, the amyloidogenic cascade is reviewed and the possible subtypes of amyloid beta are differentiated. In the second part, the roles played by the amyloid beta monomers in physiological and pathological (neurodegenerative) conditions are illustrated based on the most relevant and recent studies published on this topic. Finally, considering the importance of amyloid beta monomers in the pathophysiology of Alzheimer’s disease, new research directions with diagnostic and therapeutic impacts are suggested.

## 1. Introduction

Alzheimer’s disease (AD), the most common dementia worldwide, has been a major topic of research in the last decades [1]. Despite numerous studies conducted in view of better understanding the pathophysiology of AD, there are still many unknowns regarding the etiology of this disease. Numerous hypotheses have been proposed, including the cholinergic hypothesis [2], the mitochondrial cascade hypothesis [3], the neurovascular hypothesis [4], and many more. However, the amyloid hypothesis has remained one of the most debated theories up to the present, being the starting point in the development of biomarkers and targeted therapies [5].

According to the amyloid hypothesis, AD is the result of the pathological accumulation of amyloid beta (Aβ) peptides in the extracellular space, in the form of toxic senile plaques [6]. Mutations in the amyloid precursor protein (APP) gene were found in animal models with increased Aβ cerebral load [7], supporting this theory and opening the pathway for a myriad of studies on the topics of Aβ production, accumulation, and pathological aggregation. Aβ is considered a valuable biomarker for AD, including early diagnosis, prevention, and treatment monitoring of the disease [8]. Found also in the peripheral blood and the cerebrospinal fluid (CSF) in a significant concentration proportional to the cerebral load [9], Aβ can be easily quantified via non-invasive methods in the daily clinical setting [10].

Moreover, Aβ has also become an important therapeutic target with multiple clinical trials tried to find an effective antidementia drug by addressing amyloid depletion [11,12]. Reducing the cerebral Aβ level can be done in various ways, from enzymatic cleavage and inactivation following the binding of specific antibodies to the increase of elimination from the central or peripheral sinks via diverse methods such as plasmapheresis or dialysis [13]. Although most of the conducted studies delivered inconclusive results [14], considering Aβ clearance is the main therapeutic option for the near future, as all components of the amyloidogenic pathway are now considered potential targets.

The amyloidogenic pathway remains one of the most studied molecular pathways in human pathophysiology [15], however, it still has parts that require in-depth research (i.e., the role of the p3 peptide). One recent fact relies on the increasing importance of Aβ monomers and oligomers within the complex etiology of AD [16]. Considered for a long time without any major role in the onset and development of AD, senile plaque precursors were also demonstrated to have toxic effects at the brain level [17]. Oligomers, in particular, have been shown to be the most neurotoxic, altering neuronal signaling pathways, with the subsequent leading to synaptic dysfunction, disorganization of axonal transport, and, lastly, neuronal death [18]. Going beyond oligomers, the study of Aβ monomers has reemerged as a valuable method for the in-depth understanding of the whole amyloid plaque formation process, together with developing new therapeutic strategies that target the amyloidogenic process from the very first steps of amyloid plaque formation [19].

In the context of the absence of curative treatment for AD at present, research focused on the pathophysiology of this neurodegenerative disease (NDD) is one of the main modalities to enhance the development of more effective therapies in the near future [20]. In this regard, the aim of this article is to offer a detailed overview of the role of Aβ monomers in the pathogenesis of AD, focusing mainly on clinically relevant data obtained from the most important trials conducted in recent years. After reviewing the amyloidogenic pathway, the roles of Aβ monomers in physiological and pathological conditions, with a focus on NDDs, are highlighted. Finally, new research directions based on targeting Aβ monomers are suggested as novel diagnostic and therapeutic tools are urgently needed when considering AD. The novelty of this review relies on the way authors compare Aβ monomer behavior in physiological versus pathological conditions, discussing the role of Aβ in additional disorders besides AD.

## 2. Reviewing the Amyloidogenic Pathway

In normal conditions, APP follows the non-amyloidogenic pathway, suffering a two-step cleavage process under the action of alpha (α)-secretase and gamma (γ)-secretase complexes [21]. After the first stage of processing, APP is cleaved into two distinct fragments: the sAPPα fragment and the carboxyl-terminal fragment (CTF) [21]. sAPPα is a broadly soluble ectodomain of APP, demonstrated to have important effects in both the mature and developing brain [22]. During the early stages of central nervous system (CNS) development, sAPPα is involved in the regulation of neural stem cell proliferation [23]. In the mature brain, sAPPα performs numerous functions, being involved in processes such as neuroprotection, memory formation, and synaptic plasticity [24]. Furthermore, recent research has shown that increased levels of sAPPα may even exert therapeutic effects in brain regions affected by dementia, at least in AD mouse models [25]. Regarding the CTF, this part is processed during the second step of APP cleavage via the γ-secretase complex, generating the p3 fragment [21]. The p3 peptide suffers subsequently rapid degradation and is considered to possess no significant function [26].

However, in pathological conditions, such as AD, APP is processed via the amyloidogenic pathway, a non-physiological degradation pathway with Aβ monomers as final products [8]. Firstly, APP is cleaved under the action of beta (β)-secretase, with beta-secretase 1 (BACE1) as the main β-secretase expressed in neurons [27]. BACE1 was considered for a long time a potential therapeutic target in AD, proof being the multitude of studies conducted on BACE1 inhibitors [28,29,30]. Despite initial enthusiasm, negative results from clinical trials consisting of the inefficiency of the tested drugs on Aβ clearance together with important adverse effects such as brain atrophy or weight loss led to the present situation when BACE inhibitors are not recommended as antidementia treatment [31].

There are also other enzymes involved in this process, such as BACE2 or cathepsin B, but with a much lower expression in the CNS [32]. Despite being considered additional β-secretase, they have also become interesting targets for experimental drugs [33]. One good example is the work of Ghosh et al., where the research team has developed highly selective and potent BACE2 inhibitors, with potential utility in the treatment of diabetes mellitus type 2 [33]. Developing new drugs targeting already known key molecules and enzymes is a constant concern, proof being the research conducted in order to develop platforms for BACE2 expression, purification, and crystallization, as these platforms would allow a more rapid and efficient way to produce BACE1- and BACE2-specific inhibitors [34].

Cathepsin B, a lysosomal cysteine protease, was demonstrated to play a significant role in intracellular proteolysis, including BACE1-independent Aβ degradation [35]. Indeed, there are several aspects that link cathepsin B to the amyloidogenic pathway: on one hand, cathepsin B functions as a β-secretase, promoting the production of Aβ [35]; on the other hand, it was demonstrated to degrade Aβ via C-terminal truncation, having also a protective effect on the Aβ plaque load [36]. With heterogeneous results, a new study showed that cathepsin B is involved in both the generation and degradation of Aβ depending on its type; lysosomal cathepsin B showed a degradative behavior towards Aβ in neuroglioma cell cultures, while cathepsin B localized in the plasma membrane stimulates Aβ generation in astrocytes [37].

Another important aspect is related to the different isoforms of Aβ, with Aβ40 and Aβ42 the major ones, and the Aβ42/40 ratio a validated biomarker for AD [38]. The percentage of APP degradation via the amyloidogenic versus via the non-amyloidogenic pathway remains variable and dependent on the patient’s condition. For example, even under physiological conditions, APP can be processed via the amyloidogenic pathway, leading to a majority production (90%) of Aβ40, the more soluble form of Aβ that has demonstrated protective effects [39]. In the case of AD, the production of Aβ42 predominates, with this isoform also being more prone to aggregate into senile plaques [40].

**Figure 1:**
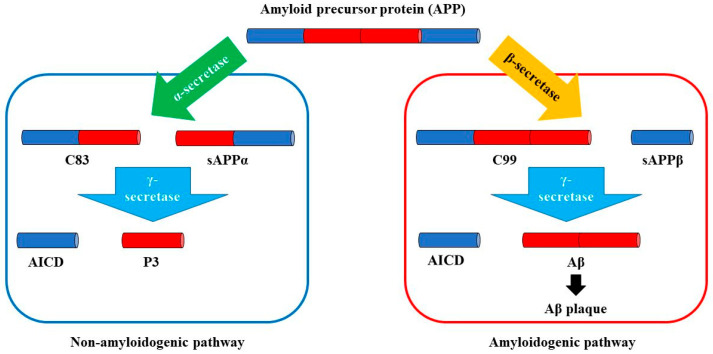
The “amyloid cascade hypothesis”—amyloidogenic versus non-amyloidogenic pathways.

As illustrated above, the amyloidogenic pathway is the main part of the “amyloid cascade hypothesis” (Figure 1), and, although studied for a long time, remains a valuable source of molecular and enzymatic targets for present and future drug development [41].

## 3. Physiological Roles of Amyloid Beta Monomers

While most studies are focused on demonstrating the pathological effects of Aβ monomers and oligomers, research has also shown beneficial roles for these compounds. Five main directions were proposed to explain the “protective” role of Aβ: antimicrobial effect, tumor suppressive activity, blood–brain barrier (BBB) sealing capacity, synaptic function regulator, and brain injury repair promoter. Each hypothesis is developed below, with Table 1 summarizing the most relevant results taken from clinical trials.

The presence of Aβ in the brain of healthy subjects has raised questions regarding the physiological roles of this compound in normal conditions. Several hypotheses were made, among the first ones being the protective capacity of Aβ against pathogens [59]. It was suggested that Aβ binds potentially toxic agents such as bacteria, viruses, and fungi, forming a complex that is subsequently presented to phagocytic cells such as macrophages and microglia [42]. The role of Aβ as an antimicrobial peptide is supported by numerous pieces of evidence in the literature. Studies conducted in vitro on cell cultures showed that Aβ can reduce the proliferation of several bacteria species [43]. This could be the result of direct mechanisms such as the formation of amyloid fibril networks that entrap pathogens, or indirect pathways that involve immune cells of the CNS. While in laboratory conditions, Aβ was proven to have protective antimicrobial effects, conclusions are difficult to draw in vivo, mainly because the concentrations of Aβ are reduced in comparison to the in vitro studies mentioned above [43].

However, the antimicrobial properties of Aβ are not restricted to bactericidal activity, as other studies demonstrated that Aβ reduced the viability of fungal in vitro cultures [44]. *Candida albicans* cultures were inhibited by homogenates obtained from the brains of AD patients, with electron microscopy showing the mechanisms of Aβ-entrapped yeast cells [45]. In a similar manner, Aβ also exerts virucidal characteristics, especially against viral pathogens with neurotropism [46]. Interesting results were obtained in research on herpes simplex virus 1, with Aβ demonstrated to inhibit the viral infection of different cell cultures as effectively as already approved antiviral agents such as acyclovir [46]. These findings are consistent proof that Aβ monomers exert protective mechanisms against a multitude of pathogenic agents of bacterial, viral, and fungal nature when aggregating in adequate amounts into more complex fibrillar networks [42,43,44,45,46].

Aβ may also be an explanation for the curious inverse correlation between AD and tumors. Many epidemiologic studies have shown that AD patients have a much lower risk of developing cancers compared to healthy subjects [60,61]. Large cohort studies confirm this relationship in the case of skin, colorectal, breast, and bladder cancers [62]. However, further research is needed to explore the mechanistic basis of this inverse relationship and to improve understanding of the molecular pathways underlying this negative correlation. While molecular pathways involving genetic mutations found in cancers (such as p53 and PIN1) are more frequently discussed when trying to explain this correlation, the intricate roles of Aβ monomers (and the impact of their assembling process) should not be forgotten. The exact mechanism that explains the Aβ tumor suppression role remains unknown. Moreover, studies suggest an indirect link between Aβ pathological aggregates and antitumoral properties [47,48,49]. Clinical trials up to the present do not support a direct mechanism of Aβ against the altered pathways encountered in different types of cancers, however, some hypotheses are related to indirect mechanisms of action. The main possible mechanisms described are related to the interception of oncogenic viruses such as human papillomavirus and Epstein–Barr virus [47], or to the inhibition of tumor cell growth mainly in tumor cell cultures in vitro [48]. Other neurotropic viruses such as human herpesvirus 6 are also considered to be related to AD pathogenesis [49], but the relationship with Aβ monomers in promoting or slowing AD onset and evolution remains to be completely explained in the near future.

Another common point between two fundamental theories of neurodegeneration is related to the Aβ–BBB bilateral interaction. The role played by BBB leakage in sustaining the neurodegenerative cascade is already known, thus indirectly increasing the Aβ load in the brain [63]. Furthermore, Aβ pathological aggregates exert their toxic effects on the cerebral milieu, having negative consequences also on the structures of the BBB [50]. However, an interesting fact is related to the potential protective function of soluble Aβ in BBB leakage. The most straightforward physical explanation is related to the capacity of Aβ filamentous aggregates to incorporate erythrocytes and other blood cells, with the tendency to seal the brain capillary walls. By interacting with red blood cells, the aggregates composed of Aβ monomers change the adherence properties of these cells, making them more adherent to the vessel’s walls [50]. This hypothesis is mainly supported by fundamental research conducted on mice models, where provoked micro-hemorrhages changed the disposition of Aβ accumulations near the sites of the lesions [51]. Other pathologies that stimulate vascular wall damage, such as diabetes and arterial hypertension, are correlated with increased Aβ cerebral load, being additional indirect evidence for the sealing capacity of the Aβ monomers.

Aβ monomers are considered to exert unknown beneficial roles in the case of traumatic brain injuries (TBI) [54]. A couple of studies showed an increased Aβ load several hours after TBI, with higher loads being correlated with better outcomes [52]. With the help of positron emission tomography (PET), clinicians were able to highlight important accumulations of Aβ in the white matter regions damaged after TBI. There is also indirect proof available, with experimental mouse models such as APP-knock-out mice showing reduced survival rates after brain injury secondary to experimental stroke provocation [53]. This relationship was explained by the hyperproduction of APP, a molecule that exerts neuroprotective effects based on its structure that ensures properties such as an increased affinity to bind heparan and the possibility to act as a growth-factor-like molecule [54]. Despite Aβ formation in the context of TBI and persistence for many years, including in the case of young patients, in the long-term, Aβ load is similar to non-injured healthy controls [64]. This confirms that Aβ exerts protective short-term effects in traumatic injuries, while the pathological accumulation encountered in AD has more complex origins.

Finally, in physiological conditions, Aβ is also involved in the regulation of synaptic activity [55,56,57,58]. Relevant brain areas such as the hippocampus are particularly dependent on the APP–Aβ turnover, with much research demonstrating the role of APP in axonal transport and the release of Aβ in the synaptic cleft during neuronal activity [65]. Aβ acts on the presynaptic neuron, stimulating neurotransmitter release, thus sustaining physiological processes that ensure the correct functioning of memory neuronal circuits [55]. This was sustained by studies conducted on rodent models for AD and other NDDs, where Aβ depletion was correlated with reduced short- and long-term memory capability [56]. Aβ, acting in physiological concentrations and durations, sustains neural plasticity by enhancing long-term potentiation and synaptic development. This phenomenon could be explained by the Aβ–acetylcholine relationship and the influence of Aβ on the quality and quantity of acetylcholine receptors [57]. Another potential explanation is based on the interaction between Aβ and the glutamate receptors [66]. In a concentration-dependent manner, Aβ can enhance glutamate receptors when in nanomolar concentrations, but can also disrupt glutamate clearance and enhance N-methyl-D-aspartate (NMDA) receptors when in picomolar concentrations [58]. This fine balance between normal and pathologically increased Aβ load and other processes occurring at the CNS level suggest that Aβ could play a variety of beneficial roles in normal conditions, however, in pathological circumstances, the excess of cerebral Aβ sustains neurodegeneration (see below).

As presented in detail previously, Aβ monomers, alone or in the form of oligomeric aggregates, perform multiple functions in physiological conditions. The antimicrobial and antitumor effects, as well as the neurorestorative capacities that remain incompletely understood, are just some of the functions of this peptide. Based on the data available in recent studies [67], Aβ monomers could be linked to other physiological processes via direct or indirect mechanisms, but this prediction must be sustained by convincing results in the future.

## 4. Amyloid Beta Monomers in Pathological Conditions

### 4.1. Amyloid Beta Monomers in Alzheimer’s Disease

Despite the fact that Aβ monomers play multiple roles in physiological conditions, this molecule was initially described in a pathological context, more precisely in AD. AD remains the most common form of dementia worldwide, and the figures regarding its prevalence and incidence are expected to at least double in the next few decades [68]. Even though it was described over 100 years ago and has benefited from a huge number of clinical and fundamental studies, scientific advances in terms of developing an effective therapy are very limited [69]. Currently, there is no curative treatment and pharmacological therapeutic means unsatisfactorily improve the clinical symptoms and the patient’s quality of life [70]. The main reason for this situation is the incomplete understanding of the disease’s etiology.

Since the first years after the detection of the first AD cases, researchers have tried to understand the pathophysiological mechanisms of this disorder [47]. Numerous processes that suffer malfunctioning have been discussed, as they are essential in generating and sustaining neurodegeneration. Proof in this sense is the multitude of currently accepted hypotheses, in spite of being only partially supported by clinical research carried out up to the present. Among the most discussed ones, worth mentioning are the chronic neuroinflammation hypothesis [71], the cholinergic hypothesis [2], the mitochondrial cascade hypothesis [3], and the neurovascular hypothesis [4]. These hypotheses are interdependent and have common elements. In addition, a common point for all the pathological processes that take place in the CNS of the AD patient is the pathological aggregation of Aβ monomers in the form of senile plaques [72]. Known as the “amyloid cascade hypothesis”, this was one of the first theories that tried to explain AD symptomatology [72]. Furthermore, pathology studies have demonstrated the veracity of this theory, as microscopically visible pathological accumulations of Aβ amyloid have been detected in AD brain samples [73]. In fact, as a model for NDDs, understanding AD offers some general principles for the theoretical comprehension of the pathological phenomena that occur within this group of heterogeneous pathologies.

From an anatomopathological perspective, NDDs are characterized by a common feature, namely the pathological accumulation of protein aggregates. There are numerous proteins that have been found to accumulate in a pathological manner intra- or extracellularly, the predominance of some compared to others being dependent on the type of NDD. Thus, in AD the accumulation of Aβ predominates, in Parkinson’s disease (PD) alpha (α)-synuclein aggregates are found more abundantly [74], while in amyotrophic lateral sclerosis (ALS), increased pathological accumulations of transactive response DNA binding protein 43 (TDP43) have been detected more recently [75]. In the clinical setting, the situation is much more complex as there are various degrees of overlap between these pathologies, including their different subtypes. An explanatory classification that demonstrates the intricate correlation between the pathological molecular findings and the heterogeneous symptomatology was provided by Allegri [76].

In pathological conditions, Aβ monomers aggregate via partially known mechanisms at the CNS level, forming neurotoxic aggregates known as senile plaques [77]. The negative impact of amyloid plaques in AD was thoroughly reviewed in several papers [77,78,79]. Aβ monomers first form oligomers, which subsequently suffer architectonic changes, transitioning from an α-helix to a β-sheet structure that encourages the toxic role of these conglomerates. In a secondary phase, oligomers form soluble protofibrils that subsequently aggregate under pathological conditions to insoluble fibrils, the basic component of amyloid plaques [80].

In contrast to its roles described in physiological conditions, in the case of NDDs, Aβ monomers have a negative impact on numerous CNS processes, supporting neurodegeneration. Whether Aβ monomers per se are considered pathological remains an open question, despite increased evidence that Aβ aggregates such as oligomers and senile plaques were demonstrated to be key factors in neurodegeneration. The current opinion in the field states that Aβ monomers’ protective or toxic impact is dependent on both concentration and isoform [81]. In this regard, while some authors have observed that neurogenesis is preferentially enhanced by Aβ40 [82], other studies have found that Aβ42 appears to favor gliogenesis [83]. Table 2 summarizes the most relevant processes influenced by the pathological Aβ aggregates.

NDDs are characterized by an important dysfunction in the interneuronal crosstalk, with synapses located in specific brain regions highly affected [84]. Synaptic dysfunction is one of the main consequences of toxic Aβ plaques and is the result of increased synaptic loss without adequate compensatory synaptogenesis [85]. This is an early AD hallmark, with synaptic damage occurring from the pre-clinical stages of AD in the CNS regions involving memory, such as the hippocampus, parahippocampal region, and amygdala [84]. The highest degree of damage was observed in the proximity of Aβ plaques, this being additional proof of the toxic microenvironment that develops around pathologically aggregated Aβ fibrils [86]. From a functional point of view, long-term potentiation is inhibited, explaining why AD patients develop memory and daily activity dysfunctionalities, despite physiological stimulation of specific neuronal circuits [87].

In a similar fashion, other structures suffer too in the nearby milieu sustained by the neurotoxic Aβ plaques. Other proteins such as Tau protein or α-synuclein express an abnormal behavior in NDDs, suffering hyperphosphorylation among other structural changes that sustain pathological aggregation. Increasing research shows that Aβ (mostly soluble mono- and oligomers) is a promotor that potentiates pathological changes in other proteic structures within the AD brain, including the cellular membrane proteins [88]. Besides the Aβ–protein bidirectional modulation, Aβ also has a direct impact on lipids, particularly on the phospholipid bilayer forming cellular membranes, explaining the negative influence of Aβ on cell integrity [89].

Intracellular processes are also negatively impacted by the pathological aggregation of Aβ monomers. According to the mitochondrial hypothesis, there is an energy-deficient balance in the AD-affected neurons, with the reduced production of adenosine 5′-triphosphate (ATP) together with the decreased mitochondrial enzymatic activity, partially explaining the neuronal loss in specific brain regions [90]. Moreover, the altered cellular homeostasis is related to impaired cellular respiration, the mitochondrial dysfunction playing a major role in glycolysis and oxygen balance [91]. Apart from mitochondrial dysfunction, other molecular pathways are also heavily altered. As a result of Aβ toxicity, calcium (Ca^2+^) influx occurs in both a glutamate receptor-dependent and non-dependent manner. Increased intracellular Ca^2+^ leads to cellular stress translated by reduced metabolic activity [92].

Finally, Aβ plays a major role in generating and sustaining neurodegeneration via indirectly modulating chronic inflammation and oxidative stress [93]. Extensive research has shown that Aβ activates the microglia and astrocytes, cells that subsequently produce inflammatory cytokines and chemokines that ensure a chronic inflammatory state [93]. Immune dysregulations are also encountered, with Aβ monomers and senile plaques considered primary targets for the immune mediator storm [94]. Inflammatory mediators act both in the sense of increasing the clearance of Aβ and eliminating other toxic species and dysfunctional synapses; however, the beneficial role is hard to be divided from its pathological impact [94]. Considering the abovementioned data, it is clear that Aβ is a key molecule in AD pathogenesis, modulating in both direct and indirect manners several processes that are disturbed in NDDs. Whether there are still other molecular pathways influenced by Aβ in AD remains a question for future research.

### 4.2. Amyloid Beta Monomers in Other Neurodegenerative Disorders

The heterogeneous group of NDDs includes other pathologies besides AD, with PD, ALS, frontotemporal dementia, and their variants posing significant socioeconomic burdens. Despite their different clinical pattern, there is an overlap of only a couple of misfolded proteins considered to be the pathogenic cause, with Aβ being one of the main key players [95]. The way Aβ is involved in the pathogenesis of the other NDDs except AD is related to Aβ’s interaction with the other pathologically modified biomarkers, such as Tau protein and α-synuclein [96].

The Aβ/Tau interaction is the most studied one, with both fundamental and clinical studies demonstrating direct and indirect relationships. Previous studies conducted on AD mouse models illustrated that Tau-targeted therapies also lead to Aβ-related cognitive deficit improvements, indirectly highlighting the toxic combination between Aβ and Tau [97]. Other models confirmed this correlation, with an increased pathological Tau aggregation in the proximity of toxic Aβ plaques [98]. There are also indirect mechanisms that explain the Aβ/Tau association, with the APOE4-mediated pathway and the pathological activation of kinases just a couple of potential mechanisms [99]. Lastly, therapies targeting primarily Aβ also showed a reduction in Tau cerebral load as a cumulative effect [100].

Another interesting interaction was observed between Aβ and α-synuclein. Studies revealed the presence of hybrid oligomers containing both proteins in the brain tissues of AD and PD patients and transgenic mouse models [101]. Mainly the mouse models have explained this interaction, with bidirectional crosstalk being observed between pathological aggregates of α-synuclein and senile plaques [102]. In vitro studies confirmed that the co-aggregation of α-synuclein and Aβ is based on their interaction, with α-synuclein monomers and oligomers enhancing the pathological aggregation of Aβ monomers in several NDDs [103]. Similar to the Aβ/Tau association, the Aβ/α-synuclein interaction occurs also via indirect mechanisms, one example being the protein phosphorylation induced by different kinases such as casein-kinase 2 and polo-like kinase 2 [104].

Finally, Aβ is of interest for any pathological entity of the NDD group as it can play the role of an early- and late-stage disease biomarker. A more complex panel consisting of Aβ, Tau, and α-synuclein could be the key to differentiating AD from other non-AD dementias, as recent studies suggest [105]. Besides searching these biomarkers in peripheral blood and the CSF, actual neuroimaging techniques, such as magnetic resonance imaging (MRI) and PET use tracers directed towards Aβ [106]. At present, three Aβ-imaging tracers have been approved by the Food and Drug Administration (FDA) for use in human brain tissue [107]. Moreover, imaging techniques have already shown their valuable input in the earlier detection of AD and other NDDs, as Aβ pathological aggregation was seen by PET imaging in clinically relevant brain regions of mild cognitive impairment patients [108]. The development of Tau-imaging tracers is also in its infancy, but preliminary studies show promising future perspectives for early diagnosis and disease monitoring [109]. Considering the number of clinical trials currently ongoing [110], there is a high chance to have more specific and valuable imaging and fluid biomarkers in the near future that will help for an earlier diagnosis of NDDs.

## 5. Conclusions and Future Research Directions

Among the currently accepted AD pathogenic hypotheses, the “amyloid cascade hypothesis” remains one of the most discussed and clinically relevant. Despite being discovered many decades ago, the amyloidogenic pathway is still a source of new findings for researchers and a potential basis for targeted treatments for clinicians. On the one hand, besides the production and aggregation of Aβ, there are other protein fragments that result after APP enzymatic cleavage and that may be significant in different physiological and pathological conditions. Furthermore, the amyloidogenic pathway also remains a source for future drug development, with specific enzymatic inhibitors already studied within clinical trials.

However, the major switch of paradigm is related to the increased focus on Aβ monomers (and oligomers), as they can become valuable therapeutic targets. Aβ monomers have been demonstrated to play major roles in physiological conditions, their antimicrobial, tumor suppressive, and neurorestorative effects, along with their capacity to regulate synaptic function being only some of their “protective” features. More beneficial roles for Aβ monomers are expected to be discovered in the near future.

Regarding pathological conditions, there are already a lot of results demonstrating the importance of Aβ monomers in AD and other NDDs, with current research highlighting the idea of overlapping of Aβ, Tau protein, α-synuclein, and the importance of their interaction for the onset and development of NDDs. Aβ monomers per se, but mainly via their aggregated forms (oligomers and senile plaques) produce much damage at the cellular and molecular levels, the negative impact being observed through mitochondrial and synaptic dysfunction, membrane disorganization, and general alteration of neuronal metabolism. The negative effect of Aβ monomers is also explained by the intricate crosstalk between Aβ and reactive oxygen species, cytokines and chemokines sustaining a chronic inflammatory state, and the modulation of other misfolded proteins.

Future research should focus more on the valorization of Aβ monomers as early AD biomarkers and/or disease monitoring in patients receiving anti-Aβ therapies. Aβ monomers could also become valuable therapeutic targets in both physiological and pathological conditions. On the one hand, the “protective” features of Aβ monomers should be enhanced in healthy subjects as prophylactic intervention, while on the other hand, highly effective drugs that target Aβ monomers when in pathological quantities should be developed. Finally, fundamental and clinical studies should also continue, as there are still many unknown and incompletely explained facts about Aβ monomers in AD and other neurological and non-neurological disorders.

## Figures and Tables

**Table 1:** The most relevant processes which explain the protective role of amyloid beta.

Process Modulated by Amyloid Beta	Proof of Hypothesis	Most Relevant References
Antimicrobial effect	Reduction of the proliferation of several bacteria species Reduction of the viability of fungi (such as *Candida albicans*) Inhibition of herpes simplex virus 1 activity	[42,43,44,45,46]
Tumor suppression	Indirect evidence Interception of oncogenic viruses Inhibition of tumor cell growth (in vitro)	[47,48,49]
Blood–brain barrier sealing	Interaction with blood cells with subsequent increase of cell-to-wall adherence Direct evidence in mouse models (provoked micro-hemorrhages correlate with Aβ depositions)	[50,51]
Promoter of nervous system repairment	Direct evidence in human in PET studies Accumulation of Aβ in areas of white matter damage	[52,53,54]
Regulation of synaptic function	Stimulation of neurotransmitter release Interaction with glutamate and NMDA receptors	[55,56,57,58]

**Table 2:** Amyloid beta impact in promoting and sustaining neurodegeneration.

Biological Process Encountered in Neurodegeneration	Amyloid Beta Impact
Synaptic dysfunction	Severe synaptic loss in the hippocampus and amygdala [84] Disturbed synaptic plasticity [85] Inhibited long-term potentiation [86,87]
Interaction with other brain proteins	Bidirectional potentiation between Aβ and Tau protein pathological aggregation [88] Interaction with cell membrane proteins [89]
Mitochondrial dysfunction	Decrease in mitochondrial enzymes [90] Compromised mitochondrial ATP production [91]
Altered calcium homeostasis	Increased cellular influx of calcium ions [92] Disturbance of calcium-related signaling pathways [92]
Chronic inflammation	Activation of microglia and astrocytes [93] Increased production of inflammatory cytokines [94] Dysregulation of immune response [94]
Oxidative stress	Increased production of reactive oxygen species [93] Dysfunctions of the antioxidant systems [91]
Altered cellular homeostasis	Impaired cellular respiration [91] Membrane dysfunction [89]

## Data Availability

All data generated or analyzed during this study are included in the published article.

## Abbreviations

AD	Alzheimer’s disease
ALS	amyotrophic lateral sclerosis
APP	amyloid precursor protein
Aβ	amyloid beta
ATP	adenosine 5′-triphosphate
BACE1	beta-secretase 1
BBB	blood–brain barrier
CNS	central nervous system
CSF	cerebrospinal fluid
CTF	carboxyl-terminal fragment
NDD	neurodegenerative disease
NMDA	N-methyl-D-aspartate
PET	positron emission tomography
TBI	traumatic brain injury
TDP43	transactive response DNA binding protein 43
